# The Expression of Glyceraldehyde-3-Phosphate Dehydrogenase Associated Cell Cycle (GACC) Genes Correlates with Cancer Stage and Poor Survival in Patients with Solid Tumors

**DOI:** 10.1371/journal.pone.0061262

**Published:** 2013-04-19

**Authors:** Dunrui Wang, Daniel R. Moothart, Douglas R. Lowy, Xiaolan Qian

**Affiliations:** 1 W2MOTIF, LLC., San Diego, California, United States of America; 2 Laboratory of Cellular Oncology, National Cancer Institute, National Institutes of Health, Bethesda, Maryland, United States of America; 3 American Qualex, Inc., San Clemente, California, United States of America; Glasgow University, United Kingdom

## Abstract

Glyceraldehyde-3-phosphate dehydrogenase (GAPDH) is often used as a stable housekeeping marker for constant gene expression. However, the transcriptional levels of *GAPDH* may be highly up-regulated in some cancers, including non-small cell lung cancers (NSCLC). Using a publically available microarray database, we identified a group of genes whose expression levels in some cancers are highly correlated with *GAPDH* up-regulation. The majority of the identified genes are cell cycle-dependent (*GAPDH* Associated Cell Cycle, or GACC). The up-regulation pattern of *GAPDH* positively associated genes in NSCLC is similar to that observed in cultured fibroblasts grown under conditions that induce anti-senescence. Data analysis demonstrated that up-regulated *GAPDH* levels are correlated with aberrant gene expression related to both glycolysis and gluconeogenesis pathways. Down-regulation of fructose-1,6-bisphosphatase (FBP1) in gluconeogenesis in conjunction with up-regulation of most glycolytic genes is closely related to high expression of *GAPDH* in the tumors. The data presented demonstrate that up-regulation of *GAPDH* positively associated genes is proportional to the malignant stage of various tumors and is associated with an unfavourable prognosis. Thus, this work suggests that GACC genes represent a potential new signature for cancer stage identification and disease prognosis.

## Introduction

Although the gene encoding glyceraldehyde-3-phosphate dehydrogenase (GAPDH) is frequently used as a stable marker for constitutive gene expression, its expression is not always constant, especially in cancer. For example, in one study of non-small cell lung cancer (NSCLC), *GAPDH* was the least stable of the 6 “reference” genes examined [1]. This housekeeping glycolytic enzyme has been implicated in multiple functions and has been found to be over-expressed in certain cancers [2].

More than 50 years ago, Warburg hypothesized that cancer growth is facilitated by tumors generating their energy through aerobic glycolysis [3]. Recent studies aimed at evaluating this hypothesis have demonstrated that cancer cells have adapted their metabolism to facilitate the uptake and incorporation of nutrients into the biomass required to produce new cells [4]. Tumor development and progression are indeed correlated with enhanced glucose uptake and/or aberrant glucose metabolism [5]–[8]. The hypoxic environment in which tumor cells reside leads to an increase in glycolytic metabolism. As a key intermediate component of glycolysis, GAPDH could serve an important role in cancer cell development and tumor progression. While it is known that most glycolytic enzymes, including GAPDH, are activated and highly expressed to respond to oxygen deprivation in the tumor [9], the role of up-regulated GAPDH in NSCLC remains unclear. In one possibly cancer-related scenario, GAPDH was found to be a pro-survival regulator of caspase-independent cell death (CICD) [10].

In the current study, a publically available microarray database was employed to identify cell cycle-dependent genes that correlate with *GAPDH* up-regulation and anti-apoptotic activity. This analysis has identified a set of cell cycle-based signature genes, designated *GAPDH* Associated Cell Cycle (GACC) genes, whose up-regulation is correlated with the aggressiveness of several tumor types and their unfavourable prognosis. Identification of GACC genes may be useful in efforts aimed at elucidating pathways that connect carbohydrate metabolism with cell cycle-based cancer cell development, which might lead to the novel cancer targets based on GACC gene expression patterns in cancers.

## Results

### Up-regulation of *GAPDH* Associated Cell Cycle (GACC) genes in non-small cell lung cancer (NSCLC)

An integrated NSCLC gene expression dataset, based on the Affymetrix GeneChip Human Genome U133 Plus 2.0 Array, was created from three independent cohorts that were directly downloaded from the publically available European Bioinformatics Institute ArrayExpress database: E-GEOD-18842, E-GEOD-19188 and E-GEOD-19804. The combined dataset, designated the full cohort, consisted of 174 NSCLCs and 156 control tissues. Preliminary analysis suggested the transcription of some cell cycle genes in the NSCLC dataset might correlate with the transcription of *GAPDH*. Given the role of GAPDH in glycolysis, this finding encouraged further gene expression profile analysis of the tumors for the relationship between carbohydrate metabolism and cell cycle regulation.

Gene expression correlation analysis identified many genes whose up-regulation in the tumors is closely correlated with *GAPDH* expression. Specifically, within the cancer cohort, 341 up-regulated genes were identified with a *GAPDH* expression correlation coefficient greater than or equal to 0.6. Of these, 117 genes (34%) are described as cell cycle-related based on the Gene Ontology biological process terminology. In the original GeneChip Human Genome U133 Plus 2.0 Array with 54,613 probes, only 2,044 related genes (3.7%) are associated with Gene Ontology biological process term “cell cycle,” which implies more than a 9-fold enrichment for cell cycle-related genes in the tumors. An even higher percentage of cell cycle-related genes (18/26, 69%; a 17-fold enrichment for cell cycle-related genes) are found when the correlation coefficient is set more stringently, at greater than or equal to 0.72 (Table 1). These genes are designated here as *GAPDH* Associated Cell Cycle (GACC) genes. The genes in the list encode proteins related to G1/S and/or G2/M phase transitions (*FOXM1, CDCA3 (Tome-1), CCNB2 (cyclin B2), BIRC5, CCNB1 (cyclin B1), CDC45, PRC1* and *CCNA2 (cyclin A2)*), and proteins involved in mitotic processes (*NCAPD2, TPX2, AURKB, PSMD2, KIF4A, KIF2C, UBE2S, and FAM83D*). *KPNA2 (importin alpha 1*) and *CDKN3* (*cyclin-dependent kinase inhibitor 3*) are also cell cycle-related. Top ranked *GAPDH* positively associated genes in this class include *triosephosphate isomerase* 1 (*TPI1*) and *glucose-6-phosphate isomerase* (*GPI*), each of which encodes a key glycolytic enzyme. Thus, only 6 of the 26 genes in the list (*CENPA*, *RAD51AP1*, *SLC2A1*, *KIF4A, RFC4, and RRM2*) may not be designated as related to the cell cycle or glycolysis. At least a sub-set of these six genes may not actually be exceptions, as CENPA is an essential centromeric protein subject to cell cycle-dependent changes [11]. There is also some evidence that suggests RAD51AP1 may contribute to neoplastic growth [12], whereas SLC2A1 is involved in glycolysis related glucose transport. Finally, KIF4A is implicated in chromosome condensation and segregation during mitosis [13], RFC4 is essential for proliferating cell nuclear antigen (PCNA) dependent DNA synthesis [14], and RRM2 encodes ribonucleotide reductase M2, which catalyzes the formation of deoxyribonucleotides from ribonucleotides in a cell-cycle dependent fashion [15]. In sum, two gene classes have been identified in the list. Class I consists of glucose metabolic pathway related genes while Class II covers cell cycle related genes.

**Table 1:** Top ranked up-regulated *GAPDH* positively associated genes in NSCLC (correlation coefficient greater than or equal to 0.72).

Gene Title	Symbol	Probe	*r* value	GO Definition	T/C	*p* value (t-test)
triosephosphate isomerase 1	TPI1	213011_s_at	0.79	glycolysis	1.09	5.06E-45
non-SMC condensinI complex, subunit D2	NCAPD2	201774_s_at	0.79	cell cycle	1.19	8.33E-35
cell division cycle associated 3	CDCA3	221436_s_at	0.78	cell cycle	1.27	3.05E-50
forkhead box M1	FOXM1	202580_x_at	0.77	cell cycle	1.54	5.89E-49
TPX2, microtubule-associated, homolog (Xenopus laevis)	TPX2	210052_s_at	0.76	cell cycle	1.62	9.96E-57
centromere protein A	CENPA	204962_s_at	0.75	centromere protein	1.59	1.29E-47
karyopherin alpha 2 (RbAG cohort 1, importin alpha 1)	KPNA2	211762_s_at	0.75	cell cycle	1.16	4.99E-43
glucose-6-phosphate isomerase	GPI	208308_s_at	0.75	glycolysis	1.11	5.81E-44
cyclin B2	CCNB2	202705_at	0.75	cell cycle	1.54	9.85E-56
cell division cycle 20 homolog (*S. cerevisiae*)	CDC20	202870_s_at	0.75	cell cycle	1.66	1.09E-57
cyclin B1	CCNB1	214710_s_at	0.74	cell cycle	1.61	2.68E-57
RAD51 associated protein 1	RAD51AP1	204146_at	0.74	DNA repair	1.32	9.97E-43
solute carrier family 2 (facilitated glucose transporter), member 1	SLC2A1	201250_s_at	0.74	glucose transport	1.45	1.02E-46
ribonucleotide reductase M2	RRM2	209773_s_at	0.74	DNA replication	1.56	2.01E-54
aurora kinase B	AURKB	209464_at	0.73	cell cycle	1.43	1.81E-44
replication factor C (activator 1) 4, 37kDa	RFC4	204023_at	0.73	DNA replication	1.24	1.91E-38
proteasome (prosome, macropain) 26S subunit, non-ATPase, 2	PSMD2	200830_at	0.73	cell cycle	1.06	2.69E-26
kinesin family member 4A	KIF4A	218355_at	0.73	microtubule-based movement	1.63	1.52E-56
baculoviral IAP repeat-containing 5	BIRC5	202095_s_at	0.73	cell cycle	1.62	5.74E-56
kinesin family member 2C	KIF2C	209408_at	0.72	cell cycle	1.42	6.65E-54
cyclin-dependent kinase inhibitor 3	CDKN3	209714_s_at	0.72	cell cycle	1.53	2.16E-54
ubiquitin-conjugating enzyme E2S	UBE2S	202779_s_at	0.72	cell cycle	1.17	3.38E-29
family with sequence similarity 83, member D	FAM83D	225687_at	0.72	cell cycle	1.40	5.67E-35
cell division cycle 45 homolog (*S. cerevisiae*)	CDC45	204126_s_at	0.72	cell cycle	1.34	5.28E-43
cyclin A2	CCNA2	203418_at	0.72	cell cycle	1.62	8.53E-49
protein regulator of cytokinesis 1	PRC1	218009_s_at	0.72	cell cycle	1.47	7.11E-51

*r* = correlation coefficient between *GAPDH* and current gene expression in NSCLC.

T/C = expression level ratio between cancer and control.

The highest ranked GACC genes are highly associated to the pathway regulated by the transcriptional factor Forkhead Box M1 (*FoxM1*, *r* = 0.77) in NSCLC. Table 2 lists the genes in the FoxM1 pathway in which their expression is related to G2/M progression, chromosomal segregation, and cytokinesis. The *r* values between the expression of a majority of these genes (19/29, 68%) and the expression of *GAPDH* in the list are above 0.6.

**Table 2:** Correlation of gene expression between *FoxM1* related genes and *GAPDH* in NSCLC.

Gene in FoxM1 pathway	Symbol	Function	*r* value	T/C	*p* value (t-test)	Probe Set ID
NDC80 homolog, kinetochore complex component (S. cerevisiae)	NDC80 (KNTC2)	Spindle check point protein	0.66	1.55	1.52E-44	204162_at
budding uninhibited by benzimidazoles 1 homolog beta (yeast)	BUB1B (BUBR1)	Spindle check point protein	0.70	1.58	1.27E-55	203755_at
polo-like kinase 1	PLK1	Spindle check point protein	0.71	1.25	3.58E-43	202240_at
MAD2 mitotic arrest deficient-like 1 (yeast)	MAD2L1	Spindle check point protein	0.71	1.57	2.29E-55	203362_s_at
budding uninhibited by benzimidazoles 1 homolog (yeast)	BUB1	Spindle check point protein	0.70	1.64	3.45E-50	209642_at
aurora kinase A	AURKA	Spindle check point protein	0.65	1.42	2.15E-54	204092_s_at
polo-like kinase 4	PLK4	Spindle check point protein	0.56	1.18	9.30E-37	204887_s_at
budding uninhibited by benzimidazoles 3 homolog (yeast)	BUB3	Spindle check point protein	0.47	1.05	3.59E-14	201457_x_at
MAD1 mitotic arrest deficient-like 1 (yeast)	MAD1L1	Spindle check point protein	0.08	1.03	1.88E-06	204857_at
centromere protein A	CENPA	FOXM target protein	0.75	1.59	1.29E-47	204962_s_at
cell division cycle 20 homolog (S. cerevisiae)	CDC20	FOXM target protein	0.75	1.66	1.09E-57	202870_s_at
aurora kinase B	AURKB	FOXM target protein	0.73	1.43	1.81E-44	209464_at
baculoviral IAP repeat-containing 5	BIRC5 (survivin)	FOXM target protein	0.73	1.62	5.74E-56	202095_s_at
NIMA (never in mitosis gene a)- related kinase 2	NEK2	FOXM target protein	0.69	1.74	1.32E-57	204641_at
centromere protein F, 350/400kDa (mitosin)	CENPF	FOXM target protein	0.67	1.47	1.01E-55	207828_s_at
cell division cycle 25 homolog B (S. pombe)	CDC25B	FOXM target protein	0.11	1.02	0.001216	201853_s_at
mitochondrial ribosomal protein 63	MRP63	FOXM target protein	0.04	0.99	0.400757	204387_x_at
antigen identified by monoclonalantibody Ki-67	MKI67	cell cycle marker proteins (KI-67)	0.67	1.40	8.04E-49	212021_s_at
cell division cycle 25 homolog C (S. pombe)	CDC25C	cell cycle marker proteins (CDC25)	0.53	1.27	3.49E-41	205167_s_at
cyclin-dependent kinase 1	CDK1	cell cycle marker proteins (CDC2)	0.70	1.39	2.03E-49	203214_x_at
cyclin B2	CCNB2	cell cycle marker proteins	0.75	1.54	9.85E-56	202705_at
cyclin B1	CCNB1	cell cycle marker proteins	0.74	1.61	2.68E-57	214710_s_at
cyclin A2	CCNA2	cell cycle marker proteins	0.72	1.62	8.53E-49	203418_at
Ubiquitin specific peptidase 22	USP22	cell cycle marker proteins	0.19	1.02	7.11E-06	216964_at
cyclin D1	CCND1	cell cycle marker proteins	0.19	0.99	0.436576	208711_s_at
CDC28 protein kinase regulatory subunit 1B	CKS1B	CDKI proteins degradation	0.64	1.17	1.09E-46	201897_s_at
S-phase kinase-associated protein 2 (p45)	SKP2	CDKI proteins degradation	0.61	1.16	1.29E-25	203625_x_at
cyclin-dependent kinase inhibitor 1B (p27, Kip1)	CDKN1B	CDK inhibitor	0.05	0.98	0.021125	209112_at

*r* = correlation coefficient between *GAPDH* and current gene expression in lung cancers.

T/C = expression level ratio between cancer and control.

One hundred twenty-three down-regulated genes, having a correlation coefficient less than or equal to −0.6, were identified in NSCLC in the course analyzing the data (data not shown). None of the genes in this group are described by the Gene Ontology biological process as cell cycle related.

In contrast to the cancer tissues, analysis of the cancer-free control tissues in the cohort identified only 5/70 (7%) of the genes that correlated positively with *GAPDH* expression (*r* greater than or equal to 0.6). The Gene Ontology biological process describes these genes as cell cycle. The correlated cell cycle genes include *CDK4* (*r* = 0.62) and *CHK1* (*r* = 0.62). These two genes were also observed to be highly expressed within the cancer tissues.

**Figure 1:**
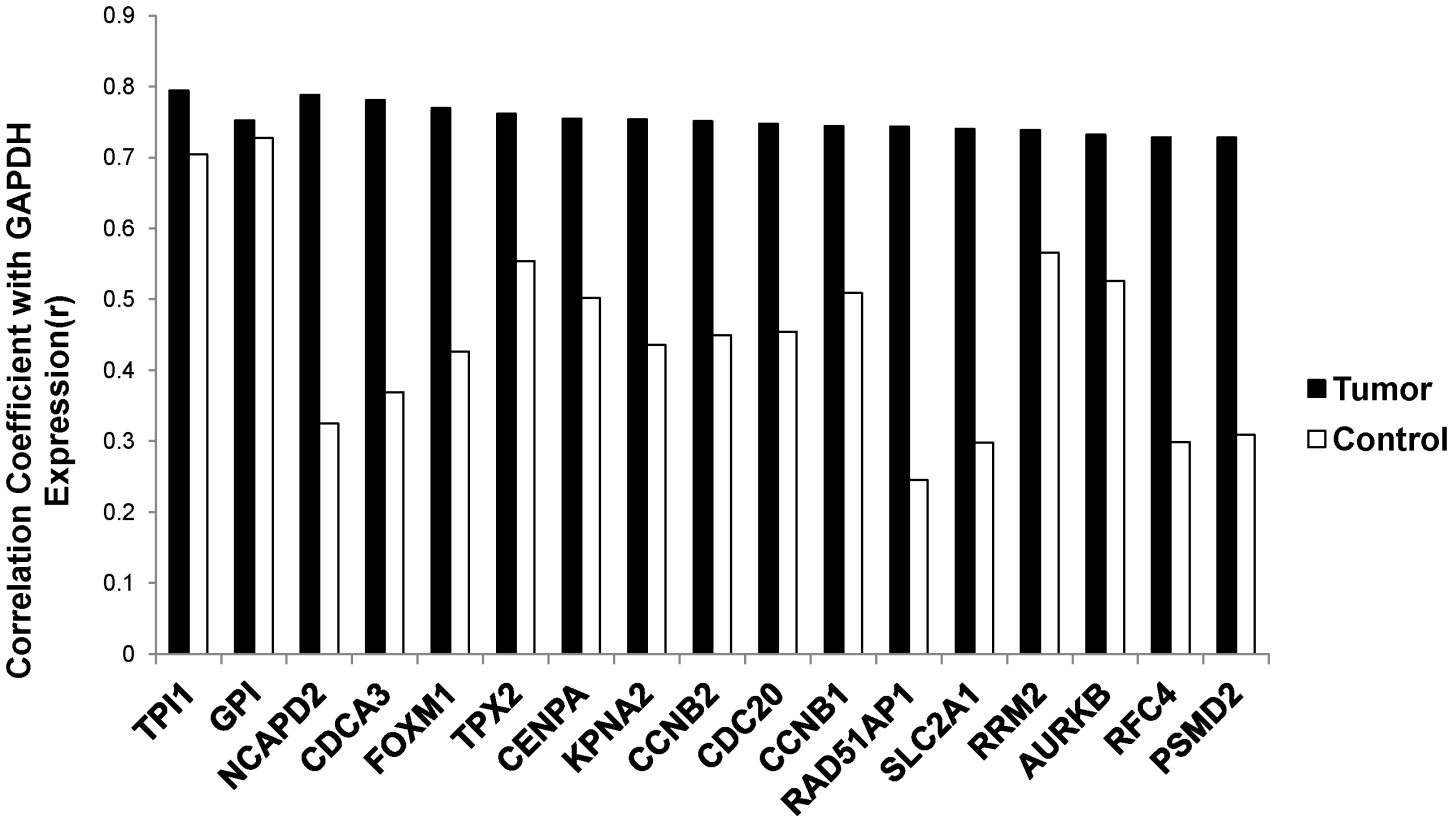
Correlation coefficients for top ranked *GAPDH* associated gene and *GAPDH* expression in NSCLCs and controls. Correlation coefficients of *GAPDH* expression levels and *GAPDH* associated gene expression levels were calculated in NSCLCs (black, selected genes with *r*>0.7) and controls (white) are plotted. *TPI1* and *GPI* are genes encoding two enzymes in glycolysis. The remainder of genes encodes cell cycle related proteins.

As implied by the above findings, for most of the highly expressed GACC genes in the tumors, there was only a weak correlation for *GAPDH* and GACC gene expression observed in the control tissue samples (correlation coefficients were mostly in 0.2–0.5 ranges). By contrast, the correlation coefficients for the 15 highest ranked GACC genes from the cancer tissues were greater than 0.7 (Figure 1). Expression of genes within the glycolytic pathway (*GAPDH*, *TPI1* and *GPI*) remained highly correlated in both in NSCLCs and non-cancer control tissue samples.

### Up-regulation of *GAPDH* in NSCLC and glycolysis/gluconeogenesis pathways

**Figure 2:**
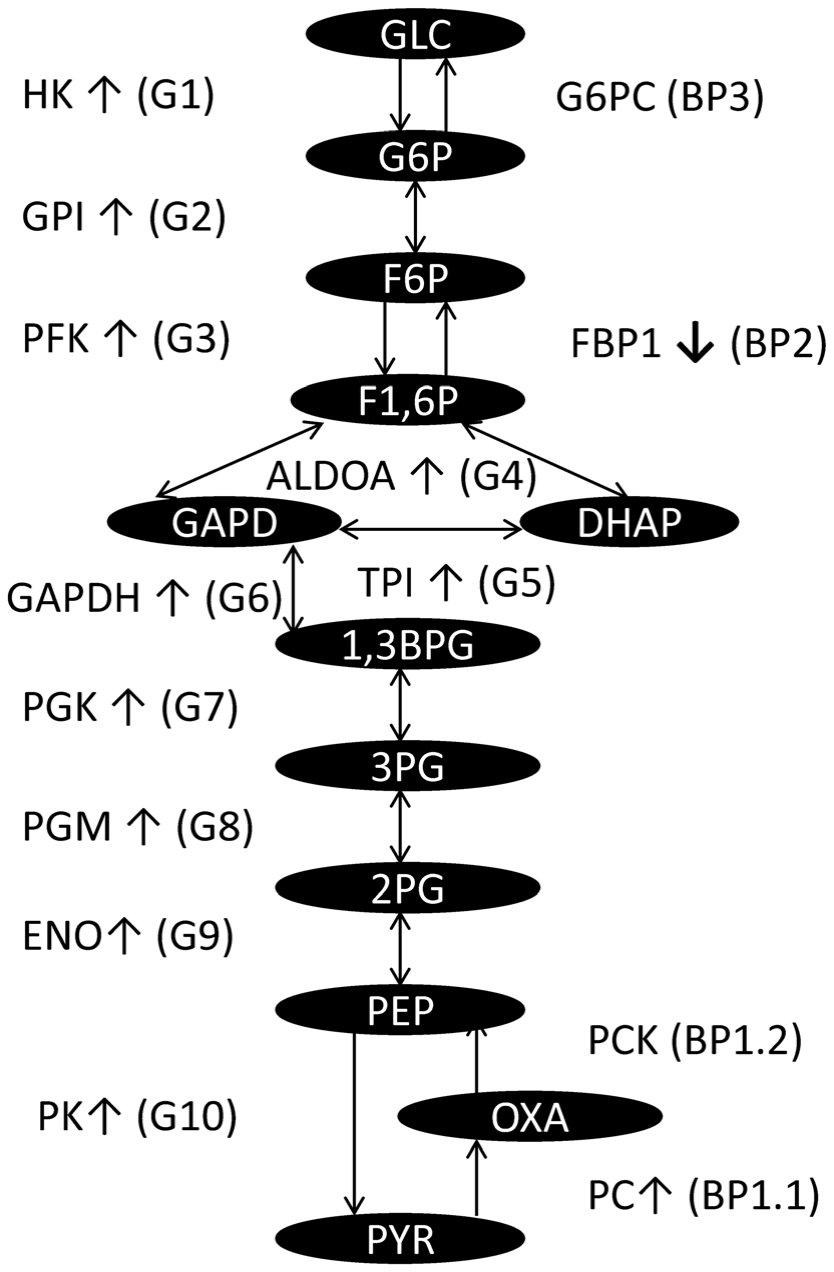
Gene expression regulation analysis of glycolysis and gluconeogenesis in NSCLC. The Figure shows the pathways of cellular glycolysis and gluconeogenesis. Oval shape text represents the metabolites of the pathways. GLC = Glucose, G6P = Glucose-6-phosphate, F6P = Fructose 6-phosphate, F1,6P = Fructose 1,6-bisphosphate, GAPD = Glyceraldehyde 3-phosphate, DHAP = Dihydroxyacetone phosphate, 1,3-BPG = 1,3-Bisphosphoglyceric acid, 3PG = Glycerate 3-phosphate, 2PG = Glycerate 2-phosphate, PEP = Phosphoenolpyruvic acid, PYR = Pyruvic acid, OXA = Oxaloacetate. Gene symbols, regulation state (↑ or ↓ if detected) and pathway (in the parentheses, G1-10: glycolysis step 1–10, BP1-3: gluconeogenesis bypass step 1–3) are indicated.

**Figure 3:**
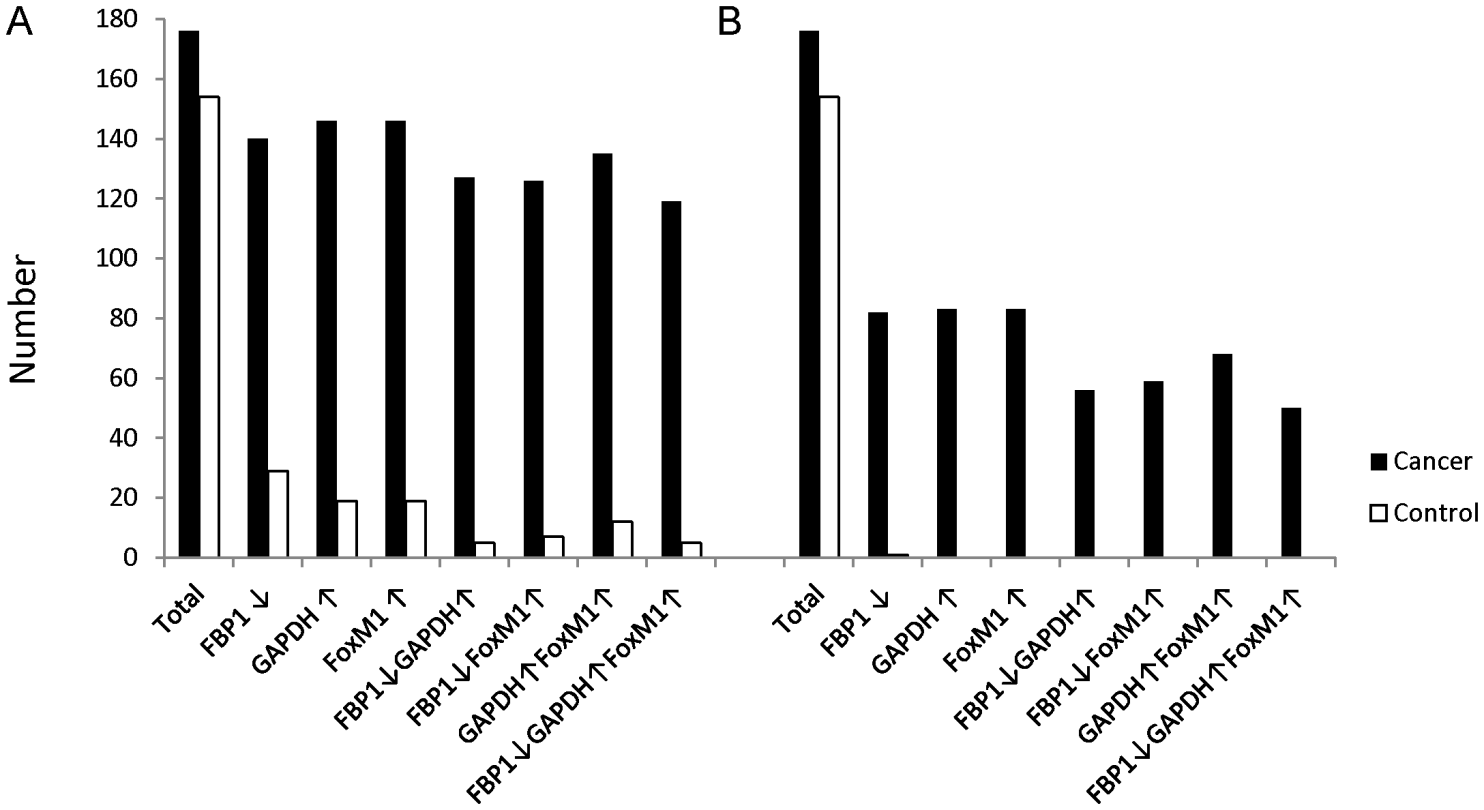
Gene expression regulation analysis of *GAPDH, FBP1 and FoxM1* in integrated NSCLC dataset. Median values (A) or 25% quartile values (B) of gene expression are considered as cut-off points. The numbers of the value higher than cut-off point (*GAPDH or FoxM1*) or lower than cut-off point (*FBP1*) or various combinations are plotted for both tumors (black) and controls (white).

The forgoing results imply that *GAPDH* expression may be associated with cell cycle related activities. To explore the relationship between the up-regulated GACC genes in lung cancer and the possibly aberrant expression of glucose metabolic pathways, we analyzed the expression values of genes implicated in glycolysis and gluconeogenesis. The expression of each gene in these pathways is up-regulated or unchanged, in the tumor cells, with the exception of fructose-1,6-bisphosphatase (*FBP1*) (Table 3, Figure 2). Up-regulation of *GAPDH* is most statistically significant among all 10 glycolysis (G1-10 in Table 3) steps and 4 bypass (BP1-3 in Table 3) gluconeogenesis steps (1.07 fold increase and one-tailed t-test *p* = 1.44E-57). Approximately 75% or 35% of NSCLC displays both increased *GAPDH* expression and decreased *FBP1* expression in the dataset using either the median or 25% quartile value as cut-off point. Furthermore, the expression of *GAPDH* and *FBP1* are co-regulated with the up-regulation of the GACC gene *FoxM1* (*r* = 0.77) in tumors (Figure 3).

**Table 3:** Gene expression of regulatory enzymes of glycolysis and gluconeogenesis.

Pathway	Gene	Symbol	T/C	*p* value (t-test)	Probe Set ID
G1	hexokinase 2	HK2	1.05	0.0007	202934_at
G2	glucose-6-phosphate isomerase	GPI	1.11	5.81E-44	208308_s_at
G3	phosphofructokinase, liver	PFKP	1.14	8.71E-35	201037_at
G4	aldolase A, fructose-bisphosphate	ALDOA	1.07	3.26E-46	200966_x_at
G5	triosephosphate isomerase 1	TPI1	1.09	1.26E-45	200822_x_at
G6	glyceraldehyde-3-phosphate dehydrogenase	GAPDH	1.07	1.44E-57	212581_x_at
G7	phosphoglycerate kinase 1	PGK1	1.05	1.75E-28	200738_s_at
G7	phosphoglycerate kinase 2	PGK2	1.05	1.65E-12	217009_at
G8	phosphoglycerate mutase family member 5	PGAM5	1.1	3.27E-24	1555943_at
G8	phosphoglycerate mutase 1 (brain)	PGAM1	1.04	1.18E-23	200886_s_at
G8	phosphoglycerate mutase 2 (muscle)	PGAM2	1.05	2.92E-07	205736_at
G9	enolase 1, (alpha)	ENO1	1.09	6.09E-45	201231_s_at
G9	enolase 2 (gamma, neuronal)	ENO2	1.11	3.71E-16	201313_at
G9	enolase 3 (beta, muscle)	ENO3	1.06	5.41E-10	204483_at
G10	pyruvate kinase, muscle	PKM2	1.09	1.50E-25	201251_at
BP-1.1	pyruvate carboxylase	PC	1.15	1.90E-32	204476_s_at
BP-1.2	phosphoenolpyruvate carboxykinase 1(soluble)	PCK1	1.01	0.0048	208383_s_at
BP-2	fructose-1,6-bisphosphatase 1	FBP1	0.84	8.45E-38	209696_at
BP-3	glucose-6-phosphatase, catalytic subunit	G6PC	1	0.1092	206952_at

G1-G10: Glycolysis step 1 to step 10. BP1-3 = gluconeogenesis bypass step 1 to step 3.

T/C = expression level ratio between cancer and control.

### Relevance of up-regulated *GAPDH* positively correlated genes in NSCLC to regulation of cell senescence

**Figure 4:**
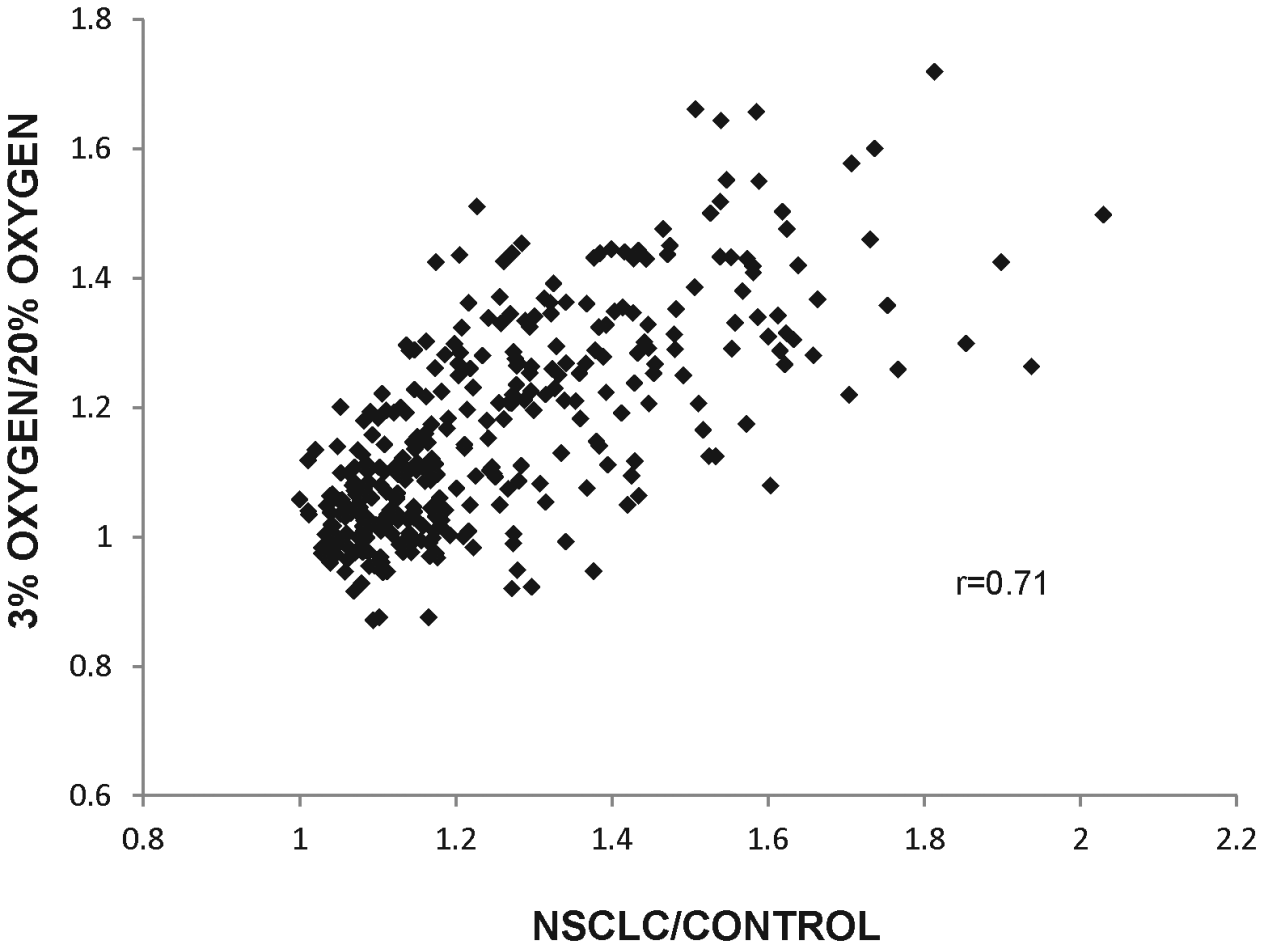
Comparison of gene expression in NSCLC and anti-senescent cells. Positive correlation coefficients between signal ratio of tumor/control in NSCLC dataset and signal ratio of 3% oxygen/20% oxygen cultured human diploid fibroblast dataset are plotted.

To further explore the correlation of the up-regulation of GACC genes and that of the other *GAPDH* positively correlated genes in NSCLC, we speculated that cancer may arise in part from a reduction in the normal regulation of senescence by genes associated with the cell cycle. We therefore examined gene expression levels in cultured cells in which anti-senescence regulation had been induced, and compared them to the genes whose up-regulated expression were found to be positively correlated with *GAPDH* expression in the NSCLC cohort. This comparison employed microarray dataset E-GEOD-19018, in which low passage IMR-90 human diploid fibroblasts had been cultured in 20% oxygen (normal replicative capacity) or 3% oxygen (increasing replicative capacity), after which RNA had been extracted and hybridized on microarrays. When the top 341 *GAPDH* positively correlated genes (*r* greater than or equal to 0.6) identified in the NSCLC dataset were compared with those from the cultured fibroblasts, the up-regulated *GAPDH* positively correlated genes (including at least 34% of the GACC genes) in both the NSCLC and the fibroblasts cultured in 3% oxygen (low oxygen tension and anti-senescence) were found to be similar (Figure 4). A good correlation (*r* = 0.71) was found between an expression signal ratio of cancer/control and the ratio of 3% oxygen/20% oxygen cultured cells in the expression of those genes.

### GACC genes are novel gene signatures for NSCLC and other solid tumors

**Figure 5:**
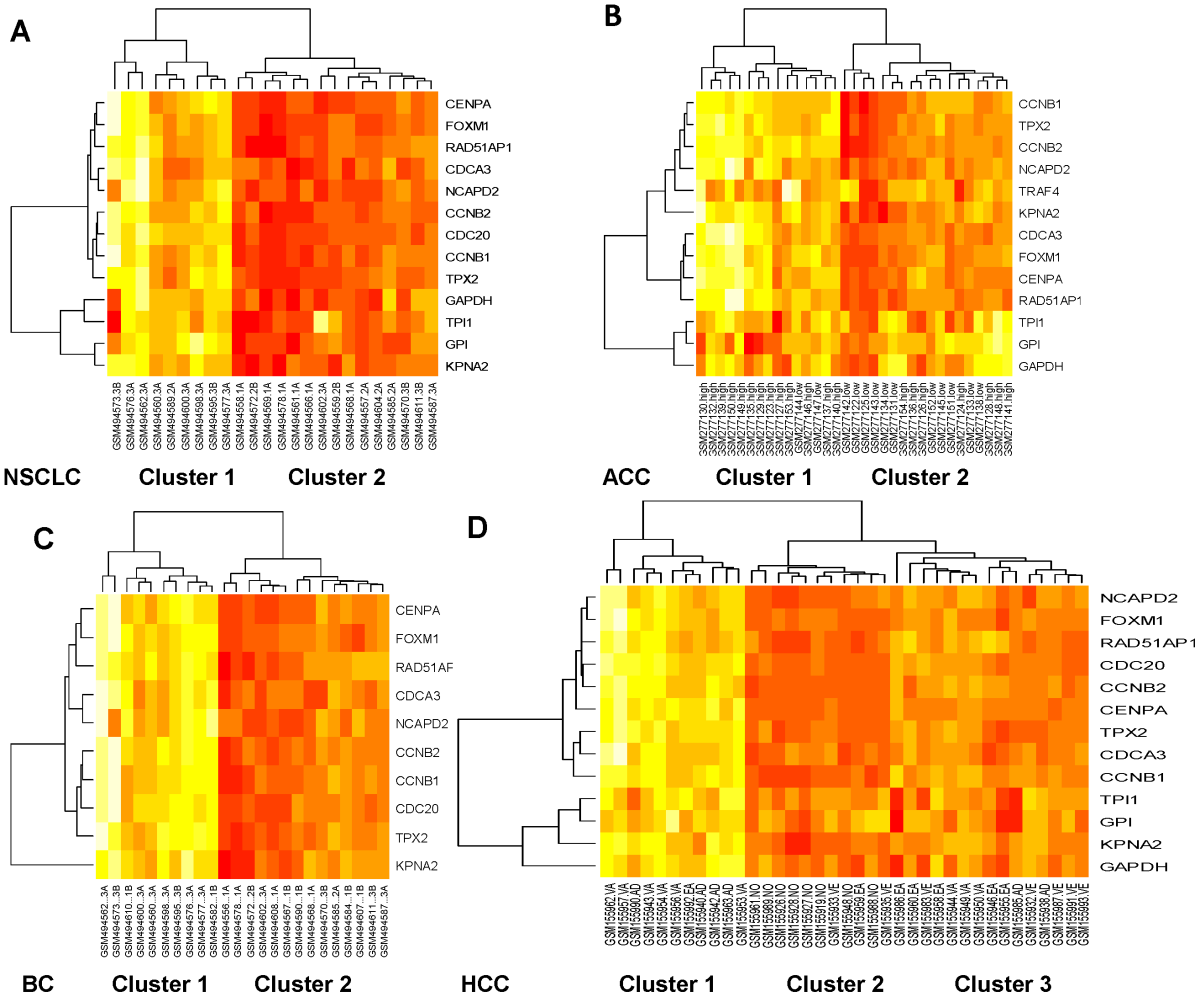
Hierarchical clustering of *GAPDH* associated genes from various cancer cohorts. The rows of a microarray heat map represent genes with each column of that row representing a different sample (source name followed by disease status). The gene expression values from four cohorts of NSCLCs with different tumor stages (I, II, III) (A), adrenocortical carcinomas (ACC) with tumor grades (high or low) (B), breast cancers (BC) with different tumor grades (I, II, III) (C), and Hepatocellular carcinomas (HCC) with different tumor stages (very early, early, advanced, very advanced) and normal liver (D) are clustered and presented by heat map.

Using various datasets available to us, we evaluated whether GACC gene expression levels may be relevant to cancer stage and prognosis. As shown in the heat map plot in Figure 5, the top ranked GACC genes, as well as the glycolysis related genes, are clustered in the tumors. Furthermore, these genes can be used to distinguish different cancer stages.

Specifically, the cohorts from E-GEOD-19804, which include 24 NSCLCs with different tumor stages (I, II, III) that had been randomly selected from the original dataset (6 stage I, 6 stage II, and 12 stage III), are preferentially clustered according to stage. Cluster 1 is preferentially associated with more advanced stage tumors, as it has 8 stage III (89%) tumors and 1 stage II tumor. By contrast, cluster 2, which has 11 stage I/II (73%) and 4 stage III tumors, is enriched for early stage tumors (Figure 5A).

The cohorts from E-GEOD-10927, which include 33 adrenocortical cancers with tumor grades (high or low), can also be clustered. Cluster 1 is enriched for high-grade tumors, as it has 13 high grade (87%) and 2 low-grade tumors. On the other hand, cluster 2 is relatively enriched for low-grade tumors, as has 11 low-grade (61%) and 7 high-grade tumors (Figure 5B).

The cohorts from E-GEOD-29431, which contain 28 breast cancers with tumor grades (I, II, III), are clustered. Fourteen stage I/II and 14 stage III samples were randomly selected from original dataset. Cluster 1 has 8 stage III (73%) and 3 stage II tumors, while cluster 2 has 11 stage I/II (65%) and 6 stage III tumors (Figure 5C).

The cohorts from E-GEOD-6764, which contain hepatocellular carcinoma (HCC) and normal liver, are clustered. Cluster 1 is composed almost entirely of very advanced/advanced III HCC (91%), while cluster 3 has mainly very early/early tumors (67%). All 8 normal samples are in cluster 2 (73%), which also includes 2 very early and 1 early tumors (Figure 5D).

**Figure 6:**
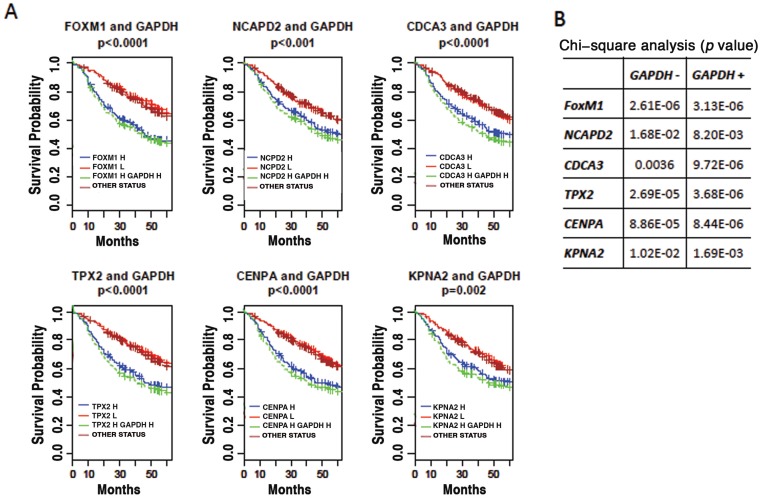
Prognostic significance of up-regulation of top ranked GACC genes and *GAPDH* in lung adenocarcinomas. (A) Director's Challenge cohort with 442 lung adenocarcinomas from caArray for Kaplan-Meier survival analysis. The up-regulation of all selected top ranked GACC gene is associated with poor prognosis. Combination of up-regulation of *GAPDH* with individual GACC gene improves the prediction power (high GACC gene expression and high *GAPDH* expression (green line) *vs* high GACC gene expression alone (blue line)). H = high. L = low. (B) Statistical significance of Kaplan-Meier survival analysis using individual GACC gene as marker (*GAPDH−*) or combination of GACC gene and *GAPDH* (*GAPDH*+) as markers. The table lists *p* values of chi-square analysis.

The pattern of gene clustering suggests that GACC related gene expression might be relevant as biomarkers to predict cancer prognosis. To evaluate this possibility, GACC gene expression levels were analyzed from a dataset of 442 lung adenocarcinomas [16] and correlated to 60-month survival outcomes. High expression of the top ranked GACC genes is associated with poor disease outcome (Figure 6A). Combining GACC gene expression level with *GAPDH* level can improve the predictive power of GACC gene level alone in most cases (Figure 6B).

## Discussion

In this study, a publically available microarray database [17] was employed to evaluate transcriptional levels of *GAPDH* and associated gene expression in solid tumors. The study data were analyzed using the Affymetrix GeneChip Human Genome U133 plus2 or U133A Array formats, which are commonly used for gene expression detection. Data analysis relied on a larger NSCLC cohort (total 330 samples) that combined independent data from various sources. Our identification of statistically significant changes in *GAPDH* expression in tumors enabled the evaluation of the gene expression profile that correlated with that of *GAPDH*.

Elevated levels of GAPDH have been observed in oncogene-induced transformation [18], angiogenesis [19] and anti-apoptotic function [20]–[22]. In other studies, however, GAPDH has been implicated in promoting apoptosis [23]–[25]. The reason for this paradox is poorly understood [26]. Barbini's work [27] has suggested that differential subcellular localization of GAPDH may contribute to its opposing biological activities in apoptotic and proliferating hepatocytes. The different functions of GAPDH may also be regulated by various levels of post-translational modification of the protein [28].

Our analysis shows that the profiles of GACC gene up-regulation are proportional to anti-senescence in the profiles in the tumors, rather than correlating with the promotion of senescence or apoptosis. We have identified a group of genes for which mRNA expression strongly correlates with *GAPDH* expression in tumor cells. In general, two such gene classes have been identified in the tumors. Class I consists of metabolic pathway related genes. Top ranked *GAPDH* positively associated genes in this class include *triosephosphate isomerase* 1 (*TPI1*) and *glucose-6-phosphate isomerase* (*GPI*), each of which encodes a key glycolytic enzyme. Class II covers cell cycle related genes that typically encode proteins involved in G2/M transition and M phase cell cycle regulation. Of these, the most informative protein appeared to be transcriptional factor Forkhead Box M1 (FoxM1), a crucial element for cell cycle regulation [29] and an important regulator of tumor metastasis [30]. *FoxM1* associated genes *CCNB2* (*r* = 0.75), *CENPA* (*r* = 0.75), *AURKB* (*r* = 0.73), *BIRC5* (*survivin r* = 0.73), *NEK2* (*r* = 0.69) are among the top GACC genes. The nuclear protein CENPF, which is a transcriptional target of FoxM1 (*CENPF*, *r* = 0.67), regulates the spindle assembly checkpoint to ensure proper chromosome stability and segregation during mitosis [31]. Up-regulation of these genes is consistently associated with high-expressions of *GAPDH*.

The mechanism by which GAPDH and FoxM1 may be co-regulated is unclear. In order to trigger cell cycle related events, it is possible that both GAPDH and FoxM1 translocate from the cytoplasm to the nucleus in cancer cells. Nuclear translocation of GAPDH may be regulated by acetylation [32]. FoxM1 is localized predominantly in the cytoplasm in late G1 and S phases. Nuclear translocation of FoxM1 occurs just before progression into the G2/M phase of the cell cycle and requires activity within the Raf/MEK/MAPK signaling pathway [33]. Both GAPDH and FoxM1 co-translocate to the nucleus during the G2/M transition phase through their interaction with other proteins. In this process, KPNA2 (importin alpha 1) may interact with GAPDH given that the correlation coefficient of *KPNA2* expression with *GAPDH* in NSCLC is 0.75.

GACC genes are up-regulated in the cells with 3% oxygen incubation, which have greater replication capacity (anti-senescence) than those cultured in 20% oxygen. This result is in agreement with recent studies demonstrating that GAPDH depletion switches human tumor cells to a senescent phenotype [34]. Rescue experiments that employed metabolic and genetic models confirmed that GAPDH has important regulatory functions that link energy metabolism and cell cycle networks. In sum, our data suggest that GAPDH serves a key role in cell cycle regulation and cell senescence during cancer cell development.

In addition to NSCLC, the up-regulation of *GAPDH* and associated genes, including GACC genes, was observed in other tumor types. The results suggest that transcription of GAPDH in tumors is an important step in cancer development, where it may contribute to increased cell cycle-related cell proliferation. This process may also include aberrant glycolysis and gluconeogenesis, as most genes in both pathways are up-regulated. However, the gluconeogenesis gene *FBP1* is down-regulated in the tumors. During normal glucose metabolism, excess GAPDH is continuously metabolized by glycolysis to pyruvic acid, which is then converted by FBP1 to fructose-1,6-bisphosphate during gluconeogenesis. In cancer, down-regulation of FBP1 can result in GAPDH accumulation within the cytoplasm and may also cause translocation of excess GAPDH to the nucleus. Down-regulation of FBP1 in cancer cells has been reported recently [35], [36]. FBP1 is considered a tumor suppressor gene in gastric cancer cells and down-regulation of FBP1 that is mediated by promoter hypermethylation is found in human hepatocellular carcinoma and colon cancer. Over-expression of GAPDH in the tumors may connect aberrant glucose metabolism with cell proliferation. However, our analysis cannot exclude the possibility that the observed up-regulation of both *GAPDH* and GACC genes may be a secondary effect of the cancer that is attributable to the high-energy demands required for rapid growth. While apoptosis is inhibited, cellular metabolism is increased and key metabolic pathways are activated. In addition, this study has been limited to measuring transcriptional levels based on the microarray data, and cannot establish a correlation between gene expression and cellular protein levels within the high metabolic environments. Further experimentation is required to address these issues.

Our data indicate that up-regulation of GACC genes within tumor cells is proportional to their malignant status, and thus can be a potential predictor of disease prognosis. Based on the *GAPDH* positively associated gene expression levels, the subclass of NSCLC, adrenocortical carcinoma, breast cancer and HCC can also be classified to various stages. The up-regulation of *GAPDH* positively associated genes correlates with more severe and/or later stages of cancer. Most importantly, using both *GAPDH* transcription and GACC gene expression levels in survival analysis greatly improve the predictive power of using GACC gene level alone in most cases.

## Methods

The Gene expression microarray analyses reported in this study employed data from ArrayExpress (http://www.ebi.ac.uk/arrayexpress) of the European Institute of Bioinformatics (EBI) and caArray (https://array.nci.nih.gov/caarray/home.action) of the National Cancer Institute (NCI), both of which are publicly available. The analyses included independent cohorts from ArrayEaxpress containing NSCLC (E-GEOD-18842, E-GEOD-19188 and E-GEOD-19804), adrenocortical cancer (E-GEOD-10927), breast cancer (E-GEOD-29431), hepatocellular carcinoma (HCC) (E-GEOD-6764), cell lines in various conditions (E-GEOD-19018) and a cohort from caArray containing lung adenocarcinomas (jacob-00182) for Affymetrix GeneChip Human Genome U133 plus 2 or U133A Arrays. The CEL files containing the raw data from each experiment were directly downloaded from the EBI or NCI website with particular accession number. Data were then normalized with CEL file quality control evaluation using 3′ Expression Arrays Robust Multi-array Analysis (RMA) from the Affymetrix software Expression Console (http://www.affymetrix.com). The normalized expression values represent the probe set intensity on a log-2 scale. The integrated NSCLC dataset from ArrayExpress include three independent NSCLC datasets for analysis, in which all CEL files from these sources were combined for RMA analysis.

The student's t-test (one-tailed) and Pearson's correlation coefficient calculation were carried out using Microsoft Excel. *P* values of t-test less than 0.05 were considered as statistically different. Chi Square test (chisq.test), heat map drawing (heatmap) and Kaplan-Meier survival analysis were carried out using the open source statistical tool R (version 2.14.1) (Supporting Information). In the gene expression analyses, the value of a selected gene expression level was compared with the median value or 25% quartile value of the gene expression in each cohort. The numbers higher or lower than the median or 25% quartile value are plotted in the results. For survival analysis, values higher or lower than median in each gene group were placed in “high”, “low”, or different combinations for the analysis. All survival times have been adjusted to months.

## Supporting Information

File S1 Data file for heat map (HCCdata.csv).
(CSV)

File S2 Data file for survival analysis (TPX2survival.csv).
(CSV)

File S3 R source code for bioinformatics analysis.
(DOCX)
