# Supplementary material for: The Expression of Glyceraldehyde-3-Phosphate Dehydrogenase Associated Cell Cycle (GACC) Genes Correlates with Cancer Stage and Poor Survival in Patients with Solid Tumors
Source: PLoS One. 2013 Apr 19;8(4):e61262. doi: 10.1371/journal.pone.0061262 (PMC3631177; doi:10.1371/journal.pone.0061262)
Supplement: File S3 — R source code for bioinformatics analysis. (DOCX) [file pone.0061262.s003.docx]

**Generating a heat map in R**

R>source("HCCheatmap.R")

The source code for HCCheatmap.R

a<-read.csv("HCCdata.csv")

row.names(a)<-a$X

a <- a[,2:38]

rc <- rainbow(nrow(a), start=0, end=.3)

cc <- rainbow(ncol(a), start=0, end=1)

b<-data.matrix(a)

heatmap(b)

**Survival analysis in R**

R> library(survival)

R> source("TPX2survival.R")

R> survdiff(formula = Surv(time, event) ~ TPX2, data = B)

R> survdiff(formula = Surv(time, event) ~ GAPDH, data = B)

R> survdiff(formula = Surv(time, event) ~ TG, data = B)

The source code for TPX2survival.R

B<-read.csv("TPX2survival.csv")

surv <- survfit(Surv(time, event) ~ TPX2, data = B)

surv2 <- survfit(Surv(time, event) ~ TG, data = B) #both TPX2 and GAPDH high = 1

plot(surv, lty = c(1,1), col=c("red","blue"),

ylab="Survival Probability",xlab="Months")

legend(0, 0.4, c("TPX2 High", "TPX2 Low","TPX2 High and GAPDH High","Other Status"), lty = c(1,1,2,2), col=c("blue","red","green","brown"), bty="n", cex=0.8)

par(new=TRUE)

plot(surv2, lty = c(2,2), col=c("brown","green"),

ylab="Survival Probability",xlab="Months")

title("TPX2 and GAPDH")
